# Duration of untreated psychosis and diagnostic delay in homeless patients with schizophrenia– a Copenhagen based clinical study

**DOI:** 10.1007/s00127-025-02957-6

**Published:** 2025-07-31

**Authors:** Rasmus Handest, Ida-Marie Mølstrøm, Mads Gram Henriksen, Julie Nordgaard

**Affiliations:** 1 Mental Health Center Amager, Copenhagen University Hospital, Gammel Kongevej 33, Copenhagen, 1610 Denmark; 2 Psychiatry East Region Zealand, Smedegade 16, Roskilde, 4000 Denmark; 3 Center for Subjectivity Research, Department of Communication, University of Copenhagen, Copenhagen, Denmark; 4 Department of Clinical Medicine, University of Copenhagen, Copenhagen, Denmark

**Keywords:** Self-disorder, Examination of anomalous Self-Experience (EASE), Psychopathology, Social functioning, Dual diagnosis, Positive and negative syndrome scale (PANSS)

## Abstract

**Purpose:**

Psychiatric disorder is a well-established risk factor for homelessness, and homelessness amplifies social, physical, and mental health risks. Yet, little is known about the extent and nature of diagnostic delays, including the duration of untreated psychosis (DUP), among homeless individuals with schizophrenia. This study aimed to address that gap by examining DUP, diagnostic delays, illness trajectories, psychopathology, and substance use in this population.

**Methods:**

We conducted an exploratory cross-sectional study of 35 homeless individuals with schizophrenia, using in-depth psychosocial interviews, standardized psychopathological assessments, and social functioning scales, incl. the Positive and Negative Syndrome Scale (PANSS) and the Examination of Anomalous Self-Experience (EASE).

**Results:**

The sample (mean age 32.6 years; 82.9% male) included 17 patients with paranoid schizophrenia and 10 with disorganized (hebephrenic) schizophrenia; 54% had a comorbid substance use disorder (SUD). The mean DUP was 15.5 years, with an average 6.7-year delay between first psychiatric contact and formal non-substance induced psychosis diagnosis. Patients with SUD showed shorter DUP compared to those without. Psychopathological measurements — including a mean PANSS score of 71.7 and a mean EASE score of 21.3 — were comparable to other schizophrenia samples, with no major differences between patients with or without SUD.

**Conclusion:**

Our findings indicate that the extraordinarily long DUP and diagnostic delays in this homeless sample are not explained by substance use, symptom profile, or schizophrenia subtype but point to systemic barriers in recognizing and managing severe mental illness. There is an urgent need to improve psychiatric services for homeless individuals with schizophrenia.

## Introduction

Schizophrenia affects roughly 0.45% of adults over 20 years [1], with a highly variable prognosis: almost one-quarter of patients achieve recovery, whereas about 40% face a poor long-term outcome [2].

A major factor in schizophrenia outcomes is the duration of untreated psychosis (DUP) [3], which includes delays in help-seeking, referral, and within-service processes such as missed or delayed diagnoses [4, 5]; longer DUP increases symptom burden, suicide risk, and social decline [6].

At the extreme end of social dysfunction, homelessness is linked to psychiatric disorders in 76% of cases in high-income countries [7], with ~ 10% having schizophrenia [7–9] and life expectancy reduced to ~ 50 years. Danish data mirror this, with 65% having mental illness, 43% a substance use disorder (SUD) [10], and a 10-year registry study showing schizophrenia in 14.2% of men and 11.6% of women, with life expectancy shortened by 21.6 and 17.4 years, respectively [11].

A systematic review shows that male gender, low education, unemployment, and childhood adversity increase homelessness risk, while psychotic or SUD more than double the odds [12]. A 10-year registry study found that nearly half of shelter users later receive a psychiatric diagnosis, most often schizophrenia or SUD [13].

Despite these figures, detailed clinical studies on homeless patients with psychiatric disorders—especially schizophrenia—are rare [14]. Caton et al. found that homeless men had higher rates of SUD, antisocial personality, and family fragmentation, while for women, reduced family support was the key differentiator. Historically, severe mental illness has been seen as a major driver of homelessness, as shown in Brandt’s 1992 Copenhagen survey [15] and Wilmanns’ landmark study over a century ago documented that ‘dementia praecox’—now known as schizophrenia—accounted for the majority of homelessness among vagrants he examined [16, 17].

Taken together, these findings highlight a significant knowledge gap concerning the clinical trajectories, delays in diagnosis, and functional outcomes among homeless patients with schizophrenia, and specifically, no studies have measured their DUP or service delays.

The aims of the study were therefore (i) to provide the first detailed description of DUP, service-delay components, and overall course of illness in homeless patients with schizophrenia; (ii) to explore precipitating pathways into homelessness; and (iii) to characterise psychopathology and current social functioning. By mapping these domains in depth, we seek to identify system gaps and clinical features that may inform earlier detection and tailored intervention for this highly marginalised group.

## Methods

### Study design and setting

The study was conducted in Copenhagen, Denmark, within the Homeless Outreach Psychiatric Service (HOPS)—a publicly funded, referral-free unit linked to the city’s general psychiatric hospitals. Services are free and non-obligatory. Psychiatric nurses conduct outreach in locations frequented by people experiencing homelessness, in close collaboration with social workers from the homeless community. Only individuals suspected of suffering from a psychotic disorder are included in HOPS [18].

The interviews in this study were held in locations chosen by participants, including clinics, shelters, or street settings.

### Participant eligibility and recruitment

The sample was a convenience sample; eligible participants were homeless patients with a suspected or confirmed diagnosis of schizophrenia spectrum disorder, and nurses at HOPS first screened new patients in HOPS to determine whether they could sustain a 30-minute conversation. Patients judged eligible were invited to an in-depth interview with RH, during which written informed consent was obtained and participation was confirmed. Recruitment proceeded consecutively for 20 months (December 2021– August 2023). Individuals under a court order for compulsory psychiatric treatment are not treated within HOPS and were therefore not included in the study.

### Clinical assessments

Author RH conducted all the interviews, which were video- or audio recorded and discussed with authors JN and MGH.

We examined the patients by a detailed psychosocial interview and a comprehensive psychopathological examination, including the Operational Criteria Checklist for Psychotic Illness and Affective Illness (OPCRIT) [19], the Schedule for Affective Disorders and Schizophrenia (SADS-L) [20], the Positive and Negative Syndrome Scale (PANSS) [21], the Examination of Anomalous Self-Experience (EASE) [22], the Scale to Assess Unawareness in Mental Disorder (SUMD) [23], and the Global Assessment of Functioning (GAF) [24]. Diagnoses adhered to the International Statistical Classification of Diseases and Related Health Problems, 10th Revision (ICD-10) criteria and guidelines [25].

For cross-study comparability, we assessed social functioning using both the Personal and Social Performance Scale (PSP) and Global Assessment of Functioning (GAF) [26]. The full psychiatric history of the patients, assessed from medical records, was also included and, when available, prison records. When appropriate and with the patients’ permission, we contacted relatives or staff in the shelters to gather further information.

If participants could not complete the full assessment, a limited battery (PANSS, SUMD, PSP and GAF) was administered.

Following most other studies [27], we calculated EASE scores based on a dichotomous rating of items as absent or present, and symptoms were required to have a trait-like character to be rated as present. All interviews began with a psychosocial history, after which GAF and PSP were rated during the first session; the psychopathological assessments were then conducted as soon as feasible.

All patients underwent thorough clinical history-taking and examination to assess if there was any suspicion of organic causes of psychiatric symptoms, and were referred to specialists such as neurologists when indicated, following general recommendations [28]. Given the high specificity and sensitivity of self-reported SUD, and the marginal benefit of additional paraclinical testing, patient statements regarding substance use were accepted as reliable [29].

Drawing on the in-depth psychopathological interview and psychosocial history, each participant’s first episode of homelessness was classified in a group discussion between all authors as most likely, not likely, or indeterminate (“grey area”) to be driven by psychiatric illness.

### Definitions

We defined sex as sex assigned at birth and onset of social problems as the point in time where these problems based on the psychosocial history, appeared severe enough to be noticeable to the patient’s family, friends, or teachers. We defined being homeless in accordance with the European Typology of Homelessness and Housing Exclusion (ETHOS), categorizing it as being either roofless, houseless, or in insecure or inadequate housing [30].

Onset of illness was defined as the time when psychiatric symptoms affected the level of social functioning or led to contact with psychiatry, and duration of untreated illness (DUI) from this time point to diagnosis of a non-substance-related psychotic disorder.

While some authors terminate DUP when effective antipsychotic treatment is first initiated, no gold-standard definition of DUP exists [6]. We defined DUP as the interval from the first psychotic symptom to the first non-substance-related psychosis diagnosis, with psychotic symptoms defined as those listed in ICD-10: hallucinations, delusions, or severe behavioural abnormalities such as gross excitement/over-activity, marked psychomotor retardation, or catatonia [25], with a severity equivalent to a PANSS score of 4 or higher [21]. We selected diagnosis as the primary endpoint because many participants had only brief or sub-therapeutic exposure to antipsychotic medication, making a treatment-based definition likely to underestimate unmet need. Furthermore, in the Danish mental healthcare system, a formal psychosis diagnosis grants access to a comprehensive bio-psycho-social care package, including sustained pharmacological treatment and, for homeless individuals, support in securing stable housing.

### Statistical analysis

All analyses were conducted in R version 4.3.2 [31], following STROBE guidelines and recommendations by Altman et al. [32, 33]. Analyses included: (i) description of the total sample; (ii) comparison of patients with and without SUD. Group differences in continuous variables were assessed using Welch’s t-test or Wilcoxon rank sum test (for non-normally distributed data, particularly DUP). Categorical variables were compared using confidence intervals calculated with the Wilson score method via OpenEpi [34].

## Results

### Participant recruitment and interview process

We included 35 patients in the study, 29 males and six females. No patients refused to participate. We initiated interviews with three additional patients, but excluded all three due to insufficient information: We lost contact with one patient, another patient experienced discomfort triggered by the interview, and a third patient was excluded for difficulties describing their experiences. Of the 35 participants, 7 (20%) did not complete the EASE interview, but 28 (80%) completed the full battery.

A high rate of ‘no-shows’ (approximately 40% of appointments) challenged the interview- and data collection process. This challenge was present even though we sought out patients in their shelters or other presumed locations and frequently reminded them of the appointments. A mean of 7 hours was spent interviewing each patient over a mean of 5.3 sessions. The interviews were further complicated by the temporary loss of contact with several participants for shorter or longer periods. On average, we conducted the interviews with each participant over a period of 91.7 days (range 3-370 days). In 25 of the 35 patients, the communication was challenged by psychotic symptoms or formal thought disorders.

### Participant demographics and diagnoses

Table 1 shows the demographic information and diagnoses of the sample. All patients fulfilled ICD-10 criteria for schizophrenia. Only two patients were diagnosed with schizophrenia prior to inclusion, and another five patients had been diagnosed with non-substance-related psychotic disorder before inclusion, and as such, the vast majority of patients were followed in HOPS and included in the study on the suspicion of schizophrenia. Nineteen out of the 35 included patients also fulfilled ICD-10 criteria for at least one SUD. The two most frequently used substances were cannabis and alcohol. Given that we did not exclude patients with a SUD, some patients were influenced by psychoactive substances during the interview period. In these cases, we did not diagnose schizophrenia unless we had a clear account of at least one month with no substance use provided by the patients, from admissions or prison records, in which the diagnostic criteria for schizophrenia were fulfilled.

**Table 1:** Sample characteristics

	Total sample *N* = 35
**Demographics**	
Age, years (mean)	32.6
Age 10–19, n	1
Age 20–29, n	16
Age 30–39, n	9
Age 40–49, n	6
Age 50–59, n	3
Sex, M/F (% male of group)	29/6 (82.9%)
*Education, n (% of sample)*	
10 years or less	19 (54.3%)
High school or equivalent	11 (31.4%)
Higher education	1 (2.9%)
Started, but not completed, university	4 (11.4%)
*Migrant status, n (% of sample)*	
1 st generation	8 (22.9%)
2nd generation	3 (8.6%)
**Diagnoses (ICD-10)**	
*Primary diagnosis*,* n (% of sample)*	
Paranoid Schizophrenia	17 (48.6%)
Hebephrenic Schizophrenia	10 (28.6%)
Simple Schizophrenia	3 (8.6%)
Undifferentiated schizophrenia	5 (14.3%)
*Secondary diagnoses*,* n (% of sample)*	
Patients with a secondary diagnosis of substance use disorder	19 (54.3%)
Patients with more than one secondary diagnosis of substance use disorder	14 (40.0%)
Substance dependency, cannabis	13 (37.1%)
Substance dependency, alcohol	7 (20.0%)
Substance dependency, cocaine	6 (17.1%)
Substance dependency, opioids	4 (11.1%)
Substance dependency, sedatives	2 (5.7%)
Substance dependency, hallucinogenics	1 (2.9%)

### Course of illness and social problems

Table 2 shows the course of illness and first signs of social problems. Psychotic symptoms were currently present in 32 of the patients, with an average DUP of 15.2 years. On average, the patients had their first contact with psychiatric services 9.4 years after their first psychotic symptoms and 16.4 years after their first symptoms of a psychiatric disorder. Seven of the 35 patients had had contact with child and adolescent mental health services, but none of them had previously received a diagnosis of a schizophrenia. Onset of social problems was at a mean age of 11 years, with one outlier with onset at age 29. For two of the eight patients whose first sign of social difficulties was criminality or violent behavior, the first crime was theft, whereas the first crime was violence for the remaining 6 patients. Onset of criminality was at the age of 10.9 years (mean). By the age of 13, however, all eight patients had performed acts of violence.

**Table 2:** Course of illness

Characteristic	Mean (SD) *N* = 35	Median (IQR) *N* = 35
Age for first symptoms, years	9.5 (7.5)	6 (6.0–11.0)
Age for first psychotic symptoms, years	16.5 (9.7)	15 (10.0–20.0)
Age of onset of social problems, years	13.0 (5.2)	13 (11.0–15.0)
Age of first episode of homelessness, years	24.5 (9.2)	20 (18.0–28.5)
Duration of first episode of homelessness, years	3.3 (4.7)	2 (0.9–4.0)
Age of onset of illness, years	13.9 (7.2)	13 (11.0–15.5)
Age of first contact to psychiatry, years	25.9 (9.8)	23 (19.0–30.5)
Age of schizophrenia diagnosis, years	32.5 (11.1)	30 (23.0–40.0)
Duration of untreated illness, years	18.6 (12.0)	16 (8.5–26.0)
Duration of untreated psychosis years	15.5 (14.4)	11 (4.8–21.0)
Psychiatric admissions number (all patients)	3.7 (10.7)	1 (0.0–2.5)
Suicide attempts number, among those with ≥ 1	4.1 (6.7)	2 (1.3–3.8)
**Initial sign of social problems**,** n (% of sample)**		
Conflicts with family	3 (8.6%)	
Criminality or violent behavior	8 (23%)	
Drop out or disengagement of education	13 (37%)	
Loosing job	1 (2.9%)	
Social Isolation	10 (29%)	

**Fig. 1:**
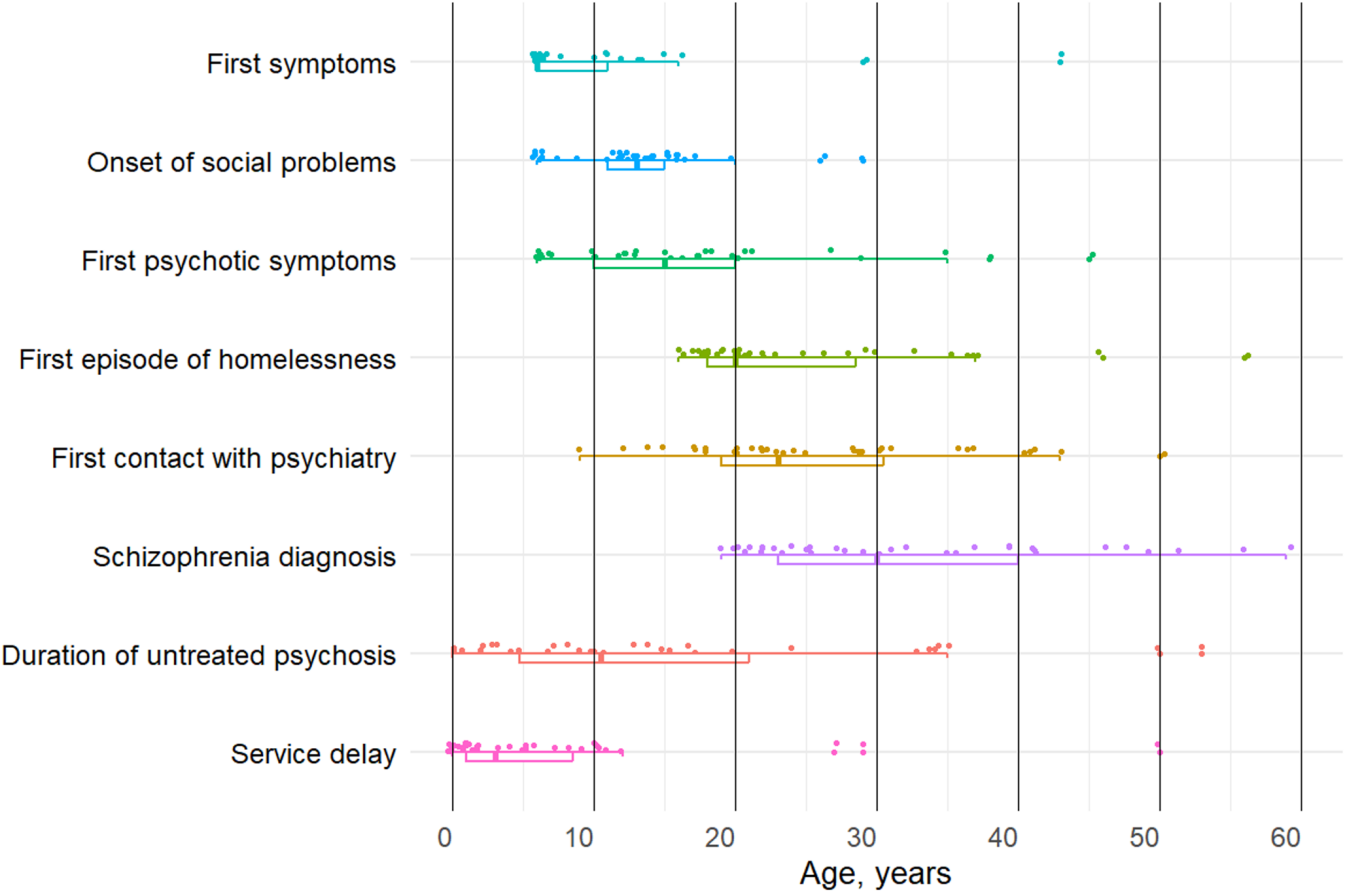
Course of illness. Combined Box-whiskers/scatter plot showing median (line), upper and lower quartile (right/left of the box), minimum/maximum (T-bars), individual values (dots) and outliers (dots outside T-bars)

Figure 1 displays selected milestones of the illness course for the patients, highlighting mean time gaps of 23 years between the first symptoms and receiving the diagnosis of schizophrenia, of 16 years between the first psychotic symptoms and the diagnosis, 8 years between the first episode of homelessness and the diagnosis, and 6.7 years between the first contact with psychiatry and the diagnosis. Twenty patients were homeless before their first contact with psychiatric services, whereas 14 had contact with psychiatric services before the first episode of homelessness. On average, social difficulties became obvious 3.5 years before the onset of psychosis.

### Psychiatric admissions, suicide attempts, and violent behaviors

In the study, 23 patients had been admitted to a psychiatric hospital, averaging 5.7 admissions each. Fourteen patients had attempted suicide, averaging 4.1 attempts per person. Additionally, 10 patients had been incarcerated, half for violent crimes, and 13 had committed violent acts at some point.

### Psychopathology and social function

Table 3 displays scores for psychopathology and social function of the sample, as well as comparison between those with and without SUD.

**Table 3:** Psychopathology, social function and duration of untreated psychosis

Scale	Total^a^	SUD: Yes^a^	SUD: No^a^	Mean difference^b^	95% CI^b, c^	*p*-value^b^
PANSS Total	71.7 (13.5)	73.9 (14.7)	69.1 (11.9)	−4.8	−4.4, 14	0.30
PANSS General	32.3 (6.8)	33.9 (6.1)	30.5 (7.2)	−3.4	−1.3, 8.1	0.15
PANSS Positive	21.3 (6.0)	21.3 (5.9)	21.3 (6.3)	0.00	−4.2, 4.2	> 0.99
PANSS Negative	18.1 (6.5)	18.7 (6.3)	17.3 (6.8)	2.0	−3.0, 6.0	0.32
EASE	21.3 (7.7)	22.3 (9.1)	20.2 (6.1)	−2.1	−4.0, 8.2	0.49
GAF-S	32.2 (7.2)	31.0 (6.4)	33.7 (8.1)	2.7	−7.8, 2.4	0.29
GAF-F	25.0 (6.1)	24.3 (4.5)	25.9 (7.7)	1.6	−6.1, 3.0	0.48
PSP	25.7 (7.5)	24.3 (5.8)	27.4 (9.1)	−2.0	−6.0, 2.0	0.31
DUP	15.5 (14.4)	10.8 (10.3)	21.6 (16.9)	−7.0	−21, 0.00	0.046

^a^Mean (SD)

^b^Welch Two Sample t-test; Wilcoxon rank sum test with continuity correction

^c^CI = Confidence Interval

SUD, Substance use disorder; PANSS, Positive and Negative Symptom Scale; EASE, Examination of Anomalous Self-Experience; GAF-F/-S, Global Assessment of Functioning– function scale/symptom scale; PSP, Personal and Social performance Scale; DUP, Duration of untreated psychosis

### Comparison of patients with and without substance use disorders

These comparisons are displayed in Table 3.

#### Causes of homelessness

The mean age for first episode of homelessness was 24.5 years, and the mean duration of the first episode was 3.3 years. The most common cause leading to the first episode of homelessness was interpersonal conflicts. Nine individuals attributed their first episode of homelessness to familial discord, whereas one patient was compelled to vacate their residence owing to association with a troublesome gang. Additionally, one patient faced homelessness after a relationship breakdown, another was forced out due to excessive noise from housemates’ parties, and yet another faced displacement following neighbor complaints stemming from disturbances in their apartment. Nine patients became homeless after having stopped paying rent, and most struggled to articulate a reason for their non-payment and simply attributed their homelessness to statements like “I stopped paying rent.” For five patients, the cause of homelessness was failure to plan for foreseeable situations, e.g., moving to another city to study without arranging a place to stay or knowing months in advance that the lease was expiring without finding another accommodation. Five patients presented non-sensical acts as reasons for their homelessness, e.g., traveling to another country without any plan, leaving the apartment due to “a bad vibe”, or thrashing the apartment “to perform financial self-harm”. Four patients did not fit the aforementioned categories: Two patients found themselves without the expected support from authorities, such as lacking accommodation post-prison release. One patient struggled to articulate a cohesive narrative regarding the circumstances leading to his initial homelessness. Furthermore, one patient’s homelessness was precipitated by apartment water damage.

#### Relationship between psychiatric disorder and homelessness

In 20 of the 35 cases, symptoms of schizophrenia were most likely the direct cause of the first episode of homelessness. In another nine cases, psychiatric disorder was perhaps the direct cause, e.g., if substance use played a major part in becoming homeless. In six cases, symptoms of psychiatric disorder were not deemed the direct cause of the first episode of homelessness, e.g., a patient cheating on his girlfriend, who then threw him out of their home, or one patient who had water damage in the apartment.

#### Current housing situation

Using the ETHOS classification for homelessness, two individuals were identified as roofless, meaning they were living rough; 22 were classified as houseless, residing in homeless shelters; eight fell into the insecure housing category, with seven couch surfing and one occupying an empty house; and two were in inadequate housing, residing in a sound studio and an allotment hut, respectively. Twelve of the patients were at some point in their lifetime rough sleepers.

## Discussion

### Delay of diagnosis and prolonged duration of untreated psychosis in homeless patients with schizophrenia

We found an alarming DUP of 15.5 years, with decades spanning from the first symptoms and initial social problems to a diagnosis of schizophrenia, and a delay of 10 years from manifestation of psychotic symptoms to the first contact with psychiatric services.

The delay we identified in this study was markedly longer than previously reported DUP figures — for instance, both a review and a Danish cohort study reported mean DUPs of only 1–2 years in patients with schizophrenia [35, 36], and similarly, a direct comparison with another group of housed patients receiving psychiatric outreach services showed considerably shorter delays [37].

#### Prior in-depth clinical examinations of homeless patients with schizophrenia

Comprehensive clinical examinations of homeless patients with schizophrenia are rare. To our knowledge, only two prior studies made such comparisons. One is the historical work by Karl Wilmanns [16], who examined 52 mentally ill, homeless individuals—all convicted of at least one crime. Most were diagnosed with dementia praecox (schizophrenia), yet their diagnoses were markedly delayed despite repeated medical evaluations during stays in prisons or workhouses. Their psychopathology was misinterpreted or overlooked for years, with first clear signs of illness appearing at 33.7 years and asylum admission at 41.3 years (based on our recalculation; Wilmanns, 1906b), thus, notably older than our sample [17]. The second study, by Caton et al. and Opler et al. [38, 39], examined 100 sheltered homeless men and 100 never-homeless controls, with a mean age similar to our sample (33.9 years). While Caton did not report on DUP, the homeless group had a reported illness onset at 20 years, which is older than the 13.9 years seen in our cohort, and first hospitalization at 21 years. The homeless group reported more positive symptoms than the controls, but the same level of negative symptoms.

While Wilmann’s study mirrored our pattern of delayed diagnosis, our findings contrast with those of Caton et al., who reported only a one-year gap between illness onset and first hospitalization. Although negative symptoms have traditionally been linked to poor social functioning [40–42], neither our study nor Caton’s found them to be more prevalent. Moreover, when we directly compared our present sample to a group of patients with schizophrenia who had stable housing but required outreach services, we found no differences in negative symptoms [43]. This aligns with recent meta-analytic evidence suggesting that all symptom domains contribute similarly to social dysfunction, indicating a broader psychopathological basis than previously assumed [44].

#### Atypical clinical presentations

While the subtypes of schizophrenia were never intended to define distinct disease entities, but rather to group symptom patterns that commonly co-occur [45], these classifications have largely fallen out of favor in contemporary psychiatry [46]. Yet, as Parnas emphasizes, abandoning these nuanced distinctions has important consequences — particularly for recognizing conditions like disorganized schizophrenia, where the distinctive Gestalt of schizophrenia often escapes the diagnostic radar.

In our sample, we observed a notably high proportion of disorganized, simple, and undifferentiated schizophrenia, with disorganized schizophrenia standing out as especially rare in other populations [47, 48]. Patients with schizophrenia often present with nonspecific or ambiguous symptoms [49], and such unclear clinical pictures can lead to diagnostic delays and prolonged DUP [4]. This challenge extends to institutional settings, where patients diagnosed with schizophrenia during incarceration face longer delays before accurate diagnosis than those diagnosed in the community prior to imprisonment — a pattern the original authors interpret as reflecting these patients’ complex, multifaceted presentations [48].

Consistent with Wilmanns’ early observations [16, 17], a part of the diagnostic delay in our sample may be attributable to these atypical symptom patterns, which required extensive, in-depth assessments to establish an accurate diagnosis—a time- and resource-intensive process that is rarely feasible in routine clinical care [50], especially for marginalized groups like the homeless. This aligns with the notion that a long DUP may not itself directly lead to a poor prognosis but may instead serve as a confounding marker, reflecting the slow and insidious onset of the disorder [51].

#### Substance use disorder and DUP

Another potential reason for the diagnostic delay could be that the SUD blurs the clinical picture. We found that SUD was present in 54% of our sample, which exceeds the prevalence reported in other samples of individuals with schizophrenia, but is comparable to other homeless populations [10, 52, 53]. However, when examining the DUP and the time from first psychiatric contact to diagnosis, we found no substantial delay in the group with SUD compared to those without; in fact, the SUD group had a shorter DUP, this finding is in keeping with a recent meta-analysis [54], and a study that reported that substance use was associated with fewer psychiatric contacts before appropriate treatment was initiated [55]. It is possible that substance use facilitated earlier contact with professionals who, in turn, helped establish links to psychiatric services.

### Relationship between psychiatric disorder and homelessness

In the majority of our sample, symptoms of psychiatric disorder were assessed to be the direct cause of the patients’ initial episode of homelessness. In contrast, only six patients did not show such a direct connection between symptoms of psychiatric disorder and becoming homeless. Yet, in these six cases, symptoms of psychiatric disorder did still contribute to the patient becoming homeless. The presence of psychiatric disorder before the first homelessness episode, potentially playing a key role in this outcome, is supported by studies of homeless patients in Copenhagen in 1992 and Heidelberg in 1906, as well as recent Danish register studies [13, 15, 16]. Early detection and treatment of symptoms of schizophrenia could minimize the risk of these vulnerable patients becoming homeless, however, even in specialized early intervention settings, homelessness is a predictor of poor prognosis [56].

### Clinical implications

The diagnostic delays and persistent homelessness described above reflect deep systemic failures in the care of this population. Homeless individuals with schizophrenia, especially those with co-occurring SUD, are frequently overlooked or deprioritized within psychiatric care [18, 57–59]. While early detection and prevention receive substantial investment, those who have already fallen through the cracks—arguably the most severely ill—are often left without proper care.

Notably, these patients are not simply disengaged. Consistent with findings from Moulin et al. [60], our results suggest that many actively seek help, often through repeated contact with emergency psychiatric services. Despite an average of seven years in contact with the system—and frequent engagement with child and adolescent services—only seven of our participants had ever received a diagnosis of a primary psychotic disorder, and just two had been diagnosed with schizophrenia. This highlights major missed diagnostic opportunities within routine care.

The consequences are severe. Nearly half of patients with comorbid schizophrenia and SUD had attempted suicide—on average five times each—and many experienced long-term homelessness and lack of recovery [14, 61]. In addition, ten participants had been incarcerated, half for violent crimes, and 13 had exhibited violent behavior from an early age. These findings are consistent with international research showing the heightened risk of imprisonment among individuals with schizophrenia, particularly when homelessness is involved [62]. This cycle of homelessness, missed care, and incarceration underscores the urgent need for more effective detection and treatment strategies for this severely underserved group.

The substantial diagnostic delay and the continuing homelessness in some of these patients despite an accurate diagnosis suggest that the psychiatric system faces major difficulties in taking care of this group of patients. This is reflected in the fact that diagnosing the patients required a team of experienced clinicians, spending several months on interviews, often lasting more than 10 h, and almost half of the appointments missed. The capacity of many current mental health services simply cannot meet these demands, which, however, seems to be needed if we are to care for and properly treat these patients.

The results from our study strongly suggest that mental health staff should be particularly attentive to homeless patients showing up in emergency room or other places within psychiatric services. At least some of the homeless patients do seem to seek help actively: Moulin et al. [60] found that patients with a primary mental health diagnosis and co-occurring SUD or homelessness were more likely to be frequent users of psychiatric emergency departments.

Diagnosing and treating a group of patients with such complex problems require specialist knowledge [18]. Unfortunately, homeless patients with schizophrenia, often with comorbid SUD, seem to be marginalized or stigmatized within mental health services [57–59]. While many resources are allocated to early detection and prevention of psychosis, few are spent on treating the persons in the streets, who have fallen through the cracks of society’s support systems, and who indisputably are among the most ill psychiatric patients of our time.

The severe delay in diagnosis and treatment has major consequences for the patients in terms of prolonged suffering, homelessness, and lack of recovery [14, 61].

### Future directions

This study exposes major gaps in diagnosing schizophrenia among people experiencing homelessness. Their extraordinarily long DUP—even after years of service contact—points to the need for mobile, outreach-based diagnostic models, especially for those with comorbid substance use. Future work should test how flexible, continuous, cross-sector services that explicitly flag homelessness as a clinical risk factor can shorten delays. Research must also clarify how diagnostic overshadowing [63]—where psychosis is masked by homelessness, substance use, behavioural problems, or insidious onset—derails timely detection, and how training clinicians in core psychopathology plus providing protected time for thorough assessments can counter this. Finally, systematic studies of structural and service barriers to early-stage care for homeless and other marginalized groups are essential.

### Limitations

The small sample size is the study’s main limitation and reflects the difficulties in obtaining data.

Although our sample included few women, the sex distribution mirrors that of Danish homeless psychiatric patients, while being slightly younger [10]. Prior research reported higher rates of SUD, non-compliance with anti-psychotic medication, and greater symptom severity in homeless males with schizophrenia; however, differences in symptom severity disappeared after adjusting for SUD and non-compliance [64]. In our study, SUD was linked to a shorter DUP, which suggests that while the male predominance may have resulted in higher SUD rates, a more balanced sex distribution would not necessarily shorten DUP and might even increase it. Nonetheless, the low female representation prevented us from performing sex-based analyses.

While the assessment of the patients involved multiple sources, including electronic health record, prison records, and input from relatives, shelter staff, and nurses, it must be acknowledged that the primary source of information was the patients themselves which entails a risk of recall bias. Not all scales, e.g., SUMD and PSP, have been validated in Danish, but they are widely used in Danish research context [65, 66], and was rated by an experienced clinician.

Due to the extensive test battery, the psychopathological rating scales were not administered on the first day, and scales such as the EASE were sometimes completed over multiple sessions.

### Conclusion

Our findings indicate that modern healthcare and support systems are poorly equipped to diagnose and care for this highly vulnerable group of patients with schizophrenia. There is a pressing need for greater attention to homeless individuals with schizophrenia—before, during, and after their contact with psychiatric services. Particular vigilance is required when homelessness is combined with key risk factors such as prior violence or criminal behavior and comorbid SUD. Finally, ensuring that clinicians have the specialized skills to recognize and diagnose the complex, often atypical clinical presentations seen in this group is essential for improving outcomes and reducing long-term harm.

## Data Availability

Raw data for the dataset are not publicly available to preserve individuals’ privacy.
